# Effect of verbal task complexity in a working memory paradigm in patients with type 1 diabetes. A fMRI study

**DOI:** 10.1371/journal.pone.0178172

**Published:** 2017-06-05

**Authors:** Joan Guàrdia-Olmos, Geisa B. Gallardo-Moreno, Esteve Gudayol-Ferré, Maribel Peró-Cebollero, Andrés A. González-Garrido

**Affiliations:** 1 Facultat de Psicologia, Universitat de Barcelona, Institut de Neurociències. Institute of Complex Systems (UBICS), Passeig de la Vall d’Hebron 171, Barcelona, Spain; 2 Instituto de Neurociencias, Universidad de Guadalajara, Francisco de Quevedo 180, Colonia Arcos Vallarta, Guadalajara, Jalisco, Mexico; 3 Facultad de Psicología, Universidad Michoacana de San Nicolás de Hidalgo, Morelia, Michoacán, Mexico; University of California, San Francisco, UNITED STATES

## Abstract

Type 1 diabetes (T1D) is commonly diagnosed in childhood and adolescence, and the developing brain has to cope with its deleterious effects. Although brain adaptation to the disease may not result in evident cognitive dysfunction, the effects of T1D on neurodevelopment could alter the pattern of BOLD fMRI activation. The aim of this study was to explore the neural BOLD activation pattern in patients with T1D versus that of healthy matched controls while performing two visuospatial working memory tasks, which included a pair of assignments administered through a block design. In the first task (condition A), the subjects were shown a trial sequence of 3 or 4 white squares positioned pseudorandomly around a fixation point on a black background. After a fixed delay, a second corresponding sequence of 3 or 4 red squares was shown that either resembled (direct, 50%) or differed from (50%) the previous stimulation order. The subjects were required to press one button if the two spatial sequences were identical or a second button if they were not. In condition B, the participants had to determine whether the second sequence of red squares appeared in inverse order (inverse, 50%) or not (50%) and respond by pressing a button. If the latter sequence followed an order distinct from the inverse sequence, the subjects were instructed to press a different button. Sixteen patients with normal IQ and without diabetes complications and 16 healthy control subjects participated in the study. In the behavioral analysis, there were no significant differences between the groups in the pure visuo-spatial task, but the patients with diabetes exhibited poorer performance in the task with verbal stimuli (p < .001). However, fMRI analyses revealed that the patients with T1D showed significantly increased activation in the prefrontal inferior cortex, subcortical regions and the cerebellum (in general p < .001). These different activation patterns could be due to adaptive compensation mechanisms that are devoted to improving efficiency while solving more complex cognitive tasks.

## Introduction

Several studies have proposed that glucose dysregulation in type 1 diabetes (T1D) can result in physiological complications, such as neuropathies, and has also been linked to an increased risk for cognitive deficits [1]. Cognitive impairments in T1D in both adults and children are reported in the areas of psychomotor speed, memory, processing speed, verbal ability, learning, attention and executive functions, including working memory [2–9]. A meta-analysis reported that patients with T1D decline in mental processing speed and mental flexibility, while the other cognitive functions seem to be spared [10]. A more recent meta-analysis by [11] suggests that adults with T1D show alterations in intelligence quotient (IQ), executive functions, memory and motor speed. However, despite the differences in these two studies, the authors of both studies concluded that the cognitive impairment in T1D ranges from mild to moderate.

Although many studies have reported that cognitive deficits in T1D are due to the effect of glycemic dysregulation on the brain [7, 12–16], some authors have postulated that T1D has a negative impact on cognition that is unrelated to the quality of metabolic control, since this variable was not associated with cognitive alterations found in T1D patients [10, 17, 18]. A previous study reported that patients with early disease onset show structural brain abnormalities that could reflect suboptimal brain development [6]. In addition, some studies suggest that the impact of T1D on brain function begins to take effect shortly after diagnosis [19, 20] and in the final stages of neurodevelopment as well [21]. Among the brain abnormalities in T1D, smaller gray matter in the left superior temporal region, right cuneus and precuneus and large gray matter volume in the right prefrontal region have been reported [22], and these changes are probably associated with both hyperglycemia and hypoglycemia [23].

Early onset T1D patients exhibit ventricular atrophy [6] and larger hippocampal volumes [24]. Diffusion tensor imaging studies with children with T1D report structural alterations in white matter that are suggestive of axonal injury or degeneration related to severe hyperglycemia [25–27]. Similarly, fMRI studies have also reported differences in the activation of brain areas in children with T1D compared to those in healthy controls [28].

As we stated earlier, despite this cerebral insult, brain adaptation to T1D could result in mild to moderate cognitive dysfunction or may not even affect cognitive functions, which some studies have reported [7]. In this sense, some works have suggested that some brain adaptations occur in T1D patients to prevent cognitive impairments. A classic paper [29] showed that in an n-back task, patients with retinopathy, compared with patients without, showed less deactivation in the anterior cingulate and the orbital frontal gyrus during hypoglycemia compared with euglycemia. However, both groups performed equally on task accuracy and reaction times. Other work [30] showed a pattern of intrinsic hyperconnectivity in children with T1D compared to that in normal controls, and they found a positive association between high connectivity patterns and good cognitive functioning in children with diabetes. In a previous work, [31] showed a similar pattern of hyperconnectivity in some patients with T1D, which was related to better information processing speed and general cognitive performance. Although a portion of these results seem to contradict those of [29], all these authors interpreted their findings in a framework of brain adaptations to prevent cognitive dysfunction in T1D.

Similarly, a study by [32] investigated the effects of acute hypoglycemia on working memory and brain function in patients with T1D, in which both patients and controls were studied using the insulin clamp technique to study both groups in two conditions: euglycemia and hypoglycemia. Their results suggest that patients with T1D present a pattern of brain activation during working memory tasks similar to the control group during a normoglycemic state, but the pattern of activation was different from that of the control subjects during hypoglycemia, and the BOLD signal in the patients with T1D was increased in areas such as the parietal and frontal cortex, the hippocampus and the cerebellum, and these patients did not deactivate several Default Mode Network areas while conducting the task, as expected. Altogether, these data suggest that, at least for some patients, interactions between T1D and working memory may exist. These support the relevance of the study of working memory and brain activation associated with this cognitive function to patients with T1D. On the other hand, the researchers that studied working memory in patients with T1D were not focused on contrasting verbal and visuospatial working memory in these patients. The study by [32] used the digit-retention task as a paradigm. Therefore, there has been no thorough study of visuospatial memory and the brain activation associated with this cognitive function in patients with T1D.

Thus, we aimed to explore neurofunctional activation in young patients with T1D during two visuospatial working memory tasks: 1) with visuospatial stimuli and 2) with the interference of verbal stimuli. There is some evidence suggesting that verbal stimuli interfere with immediate spatial memory and visuospatial working memory. Some behavioral studies have suggested that reading words presented visually interferes with immediate spatial memory [33]. On the other hand, when preparing the set of tasks in our behavioral studies, we realized that verbal stimuli were slightly interfering with working memory performance [28], and we opted for this method, given that adding stimuli caused a greater degree of interference.

We hypothesized that both groups would show activation in the prefrontal cortex, anterior cingulate and cerebellum, which have all been previously reported to be involved in working memory processing [34–36]. We also hypothesized that the aforementioned vulnerability of the T1D brain would be reflected by increased activation of the subcortical brain regions and the cerebellum in both tasks, which was previously reported [28]. In addition, similar to the results of [32] in which both groups exhibited activation of the subcortical areas and the cerebellum during a working memory task in normoglycemia, but patients with T1D exhibited much less deactivation of these areas during in hypoglycemia. Nevertheless, the second part of our hypothesis should be approached with caution because our patients with T1D were not tested during hypoglycemia. We also hypothesized that the interference of the verbal stimuli would also activate the brain regions associated with word reading and semantic processing without affecting behavioral performance in both groups.

## Materials and methods

### Participants

Patients were selected through an intentional sampling to fulfill specific inclusion criteria. The patients were recruited for this study from two associations that specialize in diabetes and the Endocrinology services from the *Centro Médico Nacional de Occidente* and *Fray Antonio Alcalde* Hospital. The institutional review board at each participating center approved the study protocol. Informed written consent was obtained from the participants or their parents or legal guardians. The entire protocol was approved by *Comité de Ética del Instituto de Neurociencias de la Universidad de Guadalajara* and also by *Comité de Enseñanza*, *Investigación y Ética del Hospital Civil de Guadalajara*.

Eligibility criteria for the T1D participants included age of onset of the disease during childhood or adolescence, right-handed, at least four years of disease evolution, normal intelligence quotient measured with the Wechsler Adult Intelligence Scale (WAIS-III), a minimum of 9 years of education and no more than two hospitalizations (due to diabetes) during the previous 2 years. Those with diabetic complications (such as retinopathy, nephropathy or neuropathy) were not included. Exclusions for both groups included neurodevelopmental disorders, neurologic or psychiatric illness and magnetic resonance imaging contraindications.

Of 22 participants for whom an fMRI scan was obtained, six were excluded due to motion-related artifacts. Sixteen T1D patients and 16 healthy control subjects matched for sex, age and education participated in the study. The patients were recruited from the endocrinology service of two hospitals and two diabetes associations from Guadalajara, Mexico. The control group was recruited from among the friends and family of the patients.

### Stimuli and procedure

During screening, the patients completed a questionnaire and provided the following information: handedness, medical history, including their last glycated hemoglobin (HbA_1c_) percentage and fasting plasma glucose levels, and current treatment or medication.

Before the fMRI session, plasma glucose was measured (Accu-Check Active glucometer). During the scanning, a working memory task (WMT) was presented. The task stimuli were administered using E-Prime Studio v.2.0 (Psychology Software Tools, Inc., 2010). The images were projected through a goggle system, and responses were collected by a magnetic-resonance compatible, hand-held, four-button response pad connected to the computer through an optical cable interface.

**Fig 1:**
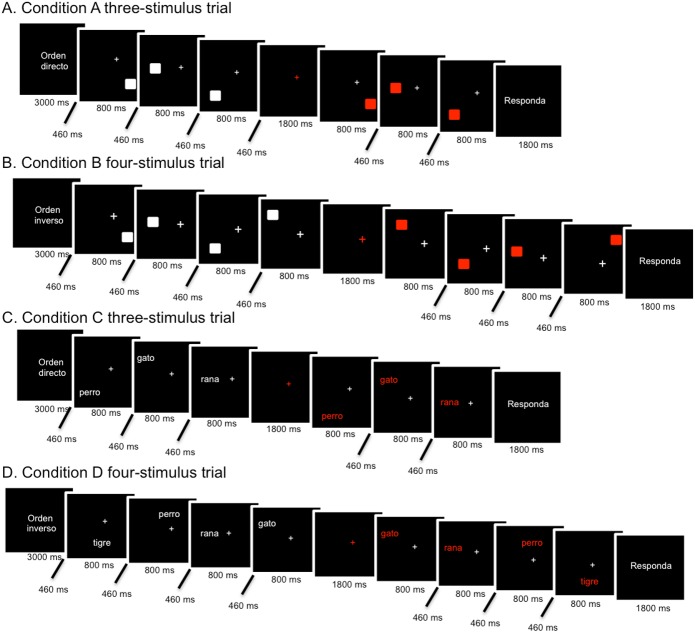
Schematic illustrations of stimulus presentation. A and B correspond to the visuospatial WM task, while C and D are part of the visuospatial verbal task. Each block of stimulus presentation was formed with a three-stimulus (A and C examples) and a four-stimulus trial (B and D examples) in all conditions.

The WMT consisted of two pairs of tasks administered in a block design. For the first task (conditions A and B, visuospatial task), the subjects were first shown a trial sequence of three (50%) or four (50%) white squares positioned pseudo-randomly around a fixation point on a black screen. In condition A, after a fixed delay following the first sequence, the subjects were shown a second sequence, which also comprised three or four red squares positioned in a direct order in 50% of the total trials (same sequence positions on the screen). In condition B, the red squares were in the inverse order in 50% of the total trials (reverse sequence positions). Then, the subjects had to press the first button (right button) if the second sequence order corresponded to exactly the same order presented before, either direct or inverse. If the red squares were in a different position from that of the white squares shown previously (either direct or inverse), then the subject had to press the second button (left button). For the second task (conditions C and D, visuospatial verbal task), common (>100 per million), familiar 2-syllable nouns were used as the stimuli instead of squares. The subjects were instructed to avoid reading and pay attention to the position and the sequence (direct in task C or inverse in task D) of the words on the screen. Then, the subjects had to perform the same tasks as A and B, in which they had to press a button if the sequence order of the red words exactly matched the order presented before with the white words, either direct or inverse. If the red words were in a different position from the white words shown previously, then subjects had to press a different button. In this task, the lexical content of the words was irrelevant to accomplish the tasks. The trial events included a 3000-ms instruction period, 800-ms stimulus presentation, 460-ms inter-interval stimuli, 1800-ms delay period and an 1800-ms response period (Fig 1). Response times and the response type (correct, incorrect or omission) were recorded for each trial.

Within a week prior to the scan session, all participants performed a series of training trials in a mock fMRI environment to familiarize them with the nature of the tasks and desensitize them to the sights and sounds that they would encounter during the scan.

### Image acquisition

A GE Excite HDxT 1.5 Tesla (General Electric Medical System, Milwaukee, WI) with a circular eight-channel head coil was used. An ultrafast three-dimensional (SPGR) image sequence was acquired. BOLD images covering the whole brain were acquired in the axial plane using an echo-planar imaging (EPI) sequence (TR/TE = 3000/60 ms; 32 slices acquired in sequential order, slice thickness = 4 mm; field of view = 25.6 cm; flip angle = 90° matrix size = 64x64). The first scans, prior to the experimental sequence, were used to localize the T1 and T2 axial series. In each experimental task, the subjects performed a total of eight blocks that lasted a total of 6:12 minutes. A total of 124 brain volumes were obtained. Due to the image acquisition time and the experimental design, 12 brain volumes per task were discarded, which left a total of 112 volumes for posterior analysis. The first two discarded volumes corresponded to the presentation of messages that helped prepare the subjects start the task. Then, before each activation block, one volume corresponding to a task instruction reminder that served as a warning to start the task was also eliminated (8 volumes in total). Finally, the last two volumes corresponded to messages indicating that the task had been completed.

### Data analyses

The demographic and behavioral results were performed using SPSS (IBM Corp. Released 2011). Analysis of variance (ANOVA) was used to assess the main and interaction effects of task condition and disease status on cognitive performance. This analysis was conducted using group (patients and controls) as the between-group factor, the four tasks (A, B, C and D) as the within-subject factor, and the percentage of correct answers and the simple response time as the dependent variables.

An fMRI analysis was carried out using the SPM8 computer package (http://www.fil.ion.ucl.ac.uk/spm/software/spm8/). Pre-statistical processing consisted of motion correction, readjustment to the voxel size, and normalization according to the MNI reference (Montreal Neurological Institute) and Talairach coordinates. For the data smoothing, a Gaussian kernel filter three times the voxel size was used on the x-, y-, z-axes (the final smoothing criteria were 12, 12 and 9 mm).

Brain activation in response to the four conditions were examined by performing a first-level general linear model (GLM) analysis for each subject using a statistical threshold of *α* = .05. To compare activation patterns between groups and conditions, a second-level full factorial GLM analysis was conducted using the same statistical threshold and applying Family Wise Error correction. All the p-values are corrected for the whole brain analysis and for a specific cluster to reduce the nominal type I error. The a posteriori effects after the main effects estimation were assessed by contrast conditions A<B to analyze the visuospatial working memory task that used squares as stimuli; the contrast C<D was also used to analyze the visuospatial working memory that used verbal stimuli; and the bilateral contrasts A-C and B-D were used to analyze the stimulus effect. A-C corresponded to immediate working memory, and B-D had the same working memory load.

## Results and discussion

In relation to the statistical description of both groups, Table 1 provides further analysis of the demographic and clinical characteristics of the participants. According to these results, we concluded that both groups are very similar in all the measured variables, with the exception of the IQ indexes (total and verbal), Verbal Comprehension Index and Processing Speed Index (p < .01).

**Table 1:** Demographic and clinical characteristics of the study subjects.

	Patients with T1D	Control subjects	Signification
*n*	16	16	No Sig.
Age (years)	20.6 (4.0)	21.13 (4.41)	No Sig.
Sex (men/women)	9/7	9/7	No Sig.
Education (years)	12.69 (2.87)	13.31 (2.75)	No Sig.
Total IQ	103.88 (7.40)	113.06 (7.30)	p < .01
Verbal IQ	100.75 (8.10)	112.13 (6.47)	p < .01
Performance IQ	107.94 (9.05)	113.44 (6.83)	No Sig.
Verbal Comprehension Index	102.88 (12.39)	116.81 (8.73)	p < .01
Perceptual Reasoning Index	109.19 (8.31)	113.44 (8.41)	No Sig.
Working Memory Index	97.00 (2.12)	99.50 (3.08)	No Sig.
Processing Speed Index	104.38 (16.36)	118.44 (10.87)	p < .01
Diabetes duration (years)	10.44 (5.37)	_	
HbA_1c_ (%)	8.91 (2.09)	_	
(mmol/mol)	74 (22.8)	_	
Last fasting plasma glucose (mg/dL)	128.54 (60.05)	_	
Plasma glucose before fMRI (mg/dL)	207.06 (72.31)	106.8 (40.19)	p < .01

The data are presented as the means (SD). n = number of cases; HbA_1c_ = glycated hemoglobin.

### Behavioral performance

For the analysis of the behavioral results of the experimental tasks, we considered only the correct responses and response times, since the percentages of incorrect responses and omissions were small and did not greatly contribute to the analysis. Initially, we estimated the linear correlations of several IQ indicators with the distributions of correct answers and reaction times. This analysis yielded no statistically significant correlations.

The descriptive results revealed a similar performance in both groups. However, we observed a slightly worse performance in the patients than in the control subjects (Table 2). However, in factorial ANOVA, this difference was not significant (Table 3). As shown in Table 3, all the reported effects have a non-significant *p*-value, with the exception of the interaction Task AB x Group (*F*(1,30) = 4.62; *p* = .040; *η*^*2*^ = .133) for correct answers and Task AB for response time (*F*(1,30) = 4.76; *p* = .037; *η*^*2*^ = .137). For the correct answers, the patients generally presented a slightly higher average value in task A than controls, whereas this effect is reversed in task B. However, this is a low-intensity effect, according to Cohen’s guidelines. In regard to the reaction time, the means were slightly higher for task A than for task B, although this effect also shows low intensity. Consequently, with the behavioral data, no relevant statistically significant differences were observed between the groups.

**Table 2:** Descriptive statistical results of task performance. Means and (Standard Deviations).

		Condition			
	Group	A	B	C	D
Correct answers (%)	Controls	88.28 (8.50)	94.53 (11.15)	92.97 (7.86)	92.19 (12.81)
	Patients	89.06 (10.07)	92.19 (11.06)	85.94 (15.05)	79.94 (16.38)
Response times (ms)	Controls	589.68 (183.64)	589.99 (149.84)	605.73 (151.56)	645.36 (158.94)
	Patients	600.19 (143.31)	590.70 (164.24)	671.95 (209.71)	649.02 (234.01)

**Table 3:** Summary results of factorial ANOVA for behavioral results.

		*F*	*df*	*p*	*η*^*2*^	1-*β*
Correct Answers	Task AB	2.52	1, 30	.123	.078	.336
	Task CD	0.09	1, 30	.765	.003	.060
	Group	3.82	1, 30	.060	.113	.473
	Task AB x Task CD	7.02	1, 30	.013	.190	.727
	Task AB x Group	4.62	1, 30	.040	.133	.548
	Task CD x Group	1.22	1, 30	.278	.039	.188
	Task AB x Task CD x Group	0.14	1, 30	.708	.005	.066
Response times	Task AB	4.76	1, 30	.037	.137	.561
	Task CD	0.03	1, 30	.869	.001	.053
	Group	0.14	1, 30	.714	.005	.065
	Task AB x Task CD	0.17	1, 30	.682	.006	.069
	Task AB x Group	0.40	1, 30	.530	.013	.094
	Task CD x Group	2.54	1, 30	.121	.078	.339
	Task AB x Task CD x Group	0.71	1, 30	.406	.023	.129

*F* = Snedecor’s *F* statistic; *p* = statistical significance; *df* = degrees of freedom; *η*^*2*^ = effect size; 1-*β* = statistical power.

### Imaging results

Given that the task comprised a visuospatial working memory task, the main effect of condition involved the right anterior prefrontal cortex and anterior cingulate, the left parahippocampal gyrus and substantia nigra, and the left anterior lobe of the cerebellum. Since tasks C and D contained verbal stimuli, activation was also found in the left inferior and middle temporal gyrus, including the fusiform gyrus and the right superior temporal gyrus next to the temporal pole.

**Fig 2:**
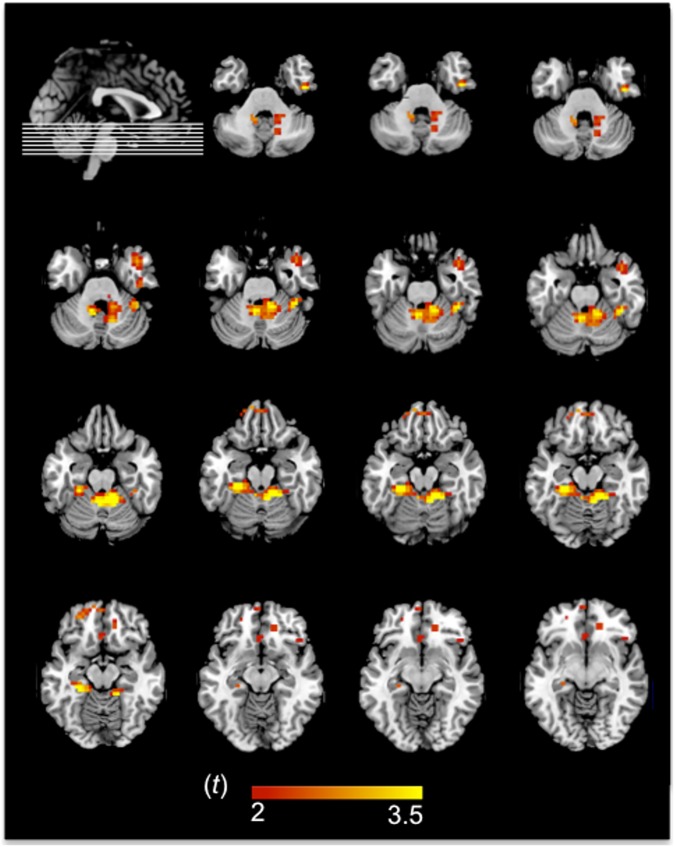
fMRI results of the interaction effect in the T1D group. Although the fMRI analyses were conducted at the whole-brain level, here we focus on the significant cluster peaks. The left hemisphere is shown on the left.

On the other hand, the group differences revealed that the patients exhibited two main subcortical activation peaks, including one peak in the right claustrum and insula and another peak in the left lentiform nucleus in the putamen. Nevertheless, we must be cautious in attributing part of this activation to a true activation of the claustrum, since the claustrum is a very small structure and was probably blurred out during the smoothing process. In addition, there were activation peaks in the right fusiform gyrus and portions of both the anterior and posterior lobes of the cerebellum in both groups. Table 4 and Fig 2 show the activated areas derived from the principal effect in ANOVA results.

**Table 4:** Statistical significance of the activations.

*F(df)*	*P*	MNI Coordinates	Cluster size(voxels)	Anatomical region		Contrasts used^1^
**Condition effect**						
5.55 (3, 120)	.001	-38 -4 -14	114	L	Inferior temporal gyrus (BA 21, 20)	Task B > A Task D > C Independent of group
4.15 (3,120)	.008	38 0–22	92	R	Superior temporal gyrus (BA 38); Claustrum	
3.53 (3,120)	.017	14 36 -6	92	R	Medial frontal gyrus (BA 45, 46, 10); Anterior cingulate (BA 32)	
3.37 (3, 120)	.021	-18 -20 -22	21	L	Parahippocampal gyrus (BA 28); Substantia nigra;	
3.09 (3, 120)	.029	-14 40 -18	4	L	Cerebellum anterior lobe (culmen)	
**Group effect**						
15.61 (1, 120)	< .001	30 4 -6	122	R	Claustrum; Insula	T1D > Control
13.5 (1, 120)	< .001	-26 8 -6	159	L	Putamen (Lentiform Nucleus)	
11.27 (1, 120)	.001	42 -24 18	87	R	Fusiform gyrus; Inferior temporal lobe (BA 20)	
9.59 (1, 120)	.002	2 -52 -22	33	B	Cerebellum anterior lobe (culmen)	
6.78 (1, 120)	.01	6 -56 -46	37	R	Cerebellum posterior lobe (tonsil)	
**Group x Condition**						
5.13 (3, 120)	.002	14 -44 -18	57	R	Cerebellum anterior lobe (culmen)	TD1 > Control
3.89 (3, 120)	.011	-26 -36 -18	19	L	Cerebellum anterior lobe (culmen)	TD1 > Control
2.95 (3, 120)	.035	14 -60 -30	7	R	Cerebellum posterior lobe (uvula)	Control > TD1
2.8 (3, 120)	.042	42 8 -30	4	R	Superior temporal gyrus (BA 38)	Control > TD1
2.74 (3, 120)	.045	-10 56 -18	4	L	Inferior frontal gyrus (BA 11, 47)	TD1 > Control

F = Snedecor’s F statistic; df = degrees of freedom; p = statistical significance; MNI coordinates = x, y, z coordinates of cluster peaks; L = left; R = right; B = bilateral; BA = Brodmann’s area.

^1^The first task/group presented more activation than the second task/group.

For the group and condition interaction effect, there was more activation in the cerebellum, particularly the culmen in the anterior lobe and the tonsil of the posterior lobe, in the T1D group in the A and B conditions. The right superior temporal gyrus (temporopolar cortex) and the left inferior frontal gyrus, specifically the orbitofrontal cortex, were more activated in the C and D conditions. On the other hand, the principal activation in the control group occurred in the right parietal lobe (precuneus) for conditions A and C and parts of the bilateral middle and superior frontal gyrus for conditions B and D.

**Fig 3:**
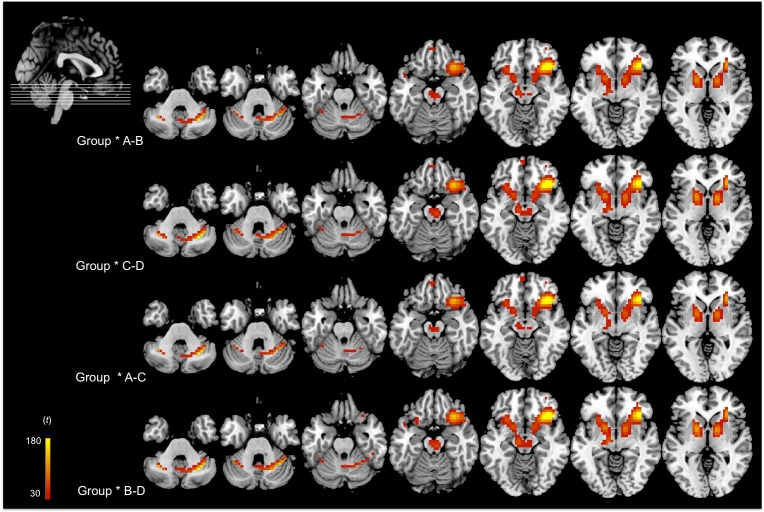
fMRI results of the interaction between group and each condition contrast. Although the fMRI analyses were conducted at the whole-brain level, here we focus on the significant cluster peaks. The left hemisphere is shown on the left.

In relation to the “a posteriori” statistical comparisons, in Fig 3 and Tables 5 and 6, we observed that for the unilateral contrasts A<B and C<D and bilateral contrast B-D the same cluster was activated, which included the right inferior frontal gyrus, specifically the orbital prefrontal cortex and the tonsil of the posterior lobe of the cerebellum, in the patients. However, in the bilateral contrast A-C, the left parahippocampal gyrus was activated in both groups.

**Table 5:** Statistical significance of a posteriori effects.

*F(df)*	*P*	MNI Coordinates	Cluster size (voxels)	Anatomical region		Contrasts used^1^
**Effect A-B**						
221.77 (2, 120)	< .001	34 24 -10	2907	R	Inferior frontal gyrus (BA 47); Cerebellum posterior lobe (tonsil)	Task B > A
**Effect C-D**						
234.89 (2, 120)	< .001	34 24 -10	2965	R	Inferior frontal gyrus (BA 47); Cerebellum posterior lobe (tonsil)	Task D > C
**Effect A-C**						
226.2 (2, 120)	< .001	34 24 -10	2807	R	Inferior frontal gyrus (BA 47); Cerebellum posterior lobe (tonsil)	Task A ≠ C
5.47 (2, 120)	.005	-22 -20 -26	12	L	Parahippocampal gyrus (BA 36)	
**Effect B-D**						
231.6 (2, 120)	< .001	38 24 -10	2803	R	Inferior frontal gyrus (BA 47); Cerebellum posterior lobe (tonsil)	Task B ≠ D

F = Snedecor’s F statistic; df = degrees of freedom; p = statistical significance; MNI coordinates = x, y, z coordinates of cluster peaks; L = left; R = right; BA = Brodmann’s area.

^1^The first task/group presented more activation than the second task/group.

**Table 6:** Statistical signification of a posteriori contrasts derived from interaction general effect.

*F(df)*	*P*	MNI Coordinates	Cluster size (voxels)	Anatomical region		Contrasts used / Group with more activation
**Group x AB**						
221.03 (2, 120)	< .001	34 24 -10	2628	R	Inferior frontal gyrus (BA 47); Cerebellum posterior lobe (tonsil)	Task B > A / TD1 > Control
27.06 (2, 120)	< .001	-58 -8 -30	53	L	Inferior temporal gyrus (BA 20)	Task B > A / Control > TD1
15.66 (2, 120)	< .001	58 -4 22	76	R	Middle temporal gyrus (BA 21)	Task B > A / Control > TD1
**Group x CD**						
235.92 (2, 120)	< .001	34 24 -10	2837	R	Inferior frontal gyrus (BA 47); Cerebellum posterior lobe (tonsil)	Task D > C / TD1 > Control
3.67 (2, 120)	.028	-26 -16 -30		L	Parahippocampal gyrus; Uncus	Task D > C / Control > TD1
**Group x AC**						
226.31 (2, 120)	< .001	34 24 -10	2751	R	Inferior frontal gyrus (BA 47); Cerebellum posterior lobe (tonsil)	Bilateral contrast / TD1 > Control
4.91 (2, 120)	.009	-26 -16 -30	13	L	Parahippocampal gyrus; Uncus; Cerebellum anterior lobe (culmen)	Bilateral contrast / Control > TD1
**Group x BD**						
231.35 (2, 120)	< .001	38 24 -10	2792	R	Inferior frontal gyrus (BA 47); Cerebellum posterior lobe (tonsil)	Bilateral contrast / TD1 > Control
25.26 (2,120)	< .001	-58 -8 -30	40	L	Inferior temporal gyrus (BA 20)	Bilateral contrast / Control > TD1

Note. *F* = Snedecor’s *F* statistic; *df* = degrees of freedom; *p* = statistical significance; MNI coordinates = x, y, z coordinates of cluster peaks; L = left; R = right; BA = Brodmann’s area.

The group x task interactions are shown in Table 6. For all contrasts in the patients with TD1, there was greater activation of the right inferior frontal gyrus, more specifically the orbitofrontal cortex, as well as the tonsil of the posterior lobe of the cerebellum. However, other clusters were also activated in the A-B contrast, such as the left inferior temporal and the right medial temporal gyri, which is closer to the ventral temporal area. Both groups showed a cluster in the left inferior temporal gyrus in the B-D contrast, while we observed greater activation of the parahippocampal gyrus and the right uncus in the C-D contrast. This same cluster was also observed in the A-C contrast, but in addition, it involved the activation of the culmen in the anterior lobe of the cerebellum.

## Discussion

In our study, we explored the neural BOLD activation pattern of patients with T1D versus a healthy matched control group while performing two visuospatial working memory tasks, which are explained in the Methods section. In the first pair of tasks AB, task A is similar to the spatial working memory task described in the study by [37] but has a higher cognitive load. Task B is very similar to task A, but the subjects had to determine whether the second sequence was exactly the reverse of the one shown previously. The tasks C and D may be considered analogous to tasks A and B, respectively, but with a verbal component that is not necessary to solve the task. Thus, these tasks also involve working memory but with a slightly higher level of difficulty and a verbal component. Therefore, our design is completely factorial, and we can therefore study the brain activations most highly associated with the verbal component in short-term memory tasks when comparing A-C, and the effect of this component on spatial memory tasks with the B-D comparison.

In our opinion, this study represents an important step towards understanding how T1D may affect the brain during cognitive activity. Multiple studies have shown that children and adults diagnosed with T1D usually achieve lower scores than their peers in different neuropsychological tests [2–9, 15, 38]. Our patients had significantly lower IQs than the control group. This finding was expected because [11] meta-analysis concluded that adults with this disease had lower IQ scores than the healthy population. Nevertheless, all the patients in our sample achieved normal IQ scores and an educational background that was significantly higher than that estimated for the overall Mexican population [39]. Therefore, the IQ difference between the groups is unlikely to cause clinically significant problems in the day-to-day activities of our T1D sample, which was previously suggested [40].

The present data demonstrated that the T1D patients showed a different functional brain activation pattern from that of the controls, as we previously reported [28]. In the present study, we used two pairs of tasks. The first pair of tasks included visuospatial working memory tasks, and the second pair of tasks included a working memory task with both visuospatial and verbal cognitive loads. As expected, for the effect of condition, regardless of the group, we found focal brain activations in the prefrontal cortex, anterior cingulate and cerebellum, which have all been previously reported to be involved in working memory processing [41, 35, 36]. Additionally, the activations observed in the left fusiform gyrus and the right temporal pole suggest that differences between the conditions (A and B versus C and D) primarily involve word recognition and semantic processing [42–44].

The a posteriori effects between conditions showed the same activation clusters in all the conducted contrasts. The clusters in the inferior prefrontal cortex and the tonsil of the cerebellum could be involved in storage processing [36, 45], which suggests that tasks B and D require more storage resources than tasks A and C. Moreover, the cerebellum may support the executive control processes [41] needed in these tasks with increasing cognitive demand. However, in the A-C contrast, there was also one more activation cluster in the left parahippocampal gyrus. Most likely, tasks A and C, which are tasks of immediate short-term memory, were easier and required fewer cognitive resources for completion. Thus, the familiarity effect (given that a square is a common shape and the words used are highly frequent in the Spanish language) may be involved in solving these tasks and could explain the left parahippocampal activations [46, 47].

When we analyzed the differences between the groups, the patients with T1D showed a different brain activation pattern with both pairs of tasks from that of the controls. In our study, regarding the behavioral performance in the tasks with verbal stimuli versus the tasks with only visuospatial stimuli, the patients with T1D performed worse in tasks with verbal stimuli. Although the participants were explicitly instructed to avoid reading, the results suggest that, probably due to reading automation in adults, both the position of the visually presented words on the screen and the lexical and semantic content of the words were encoded [48]. In addition, the participants could use verbal coding to remember the positions on the screen (e.g., “top left”). According to [49], visuospatial memory should be susceptible to interference from verbal material. In this case, the verbal stimuli presented in our tasks could elicit both the central executive processes and the focus of attention to try to maintain additional verbal information [50].

The load theory [51] claims that the perceptual load reduces distractor interference, whereas working memory load increases interference. However, recent studies suggest that the interactive effects of the working memory load and the perceptual load depend on the relationship between the modalities of working memory and stimuli [52, 53]. In our study, although the lexical content of the words was irrelevant to accomplish the C and D tasks, an additional automatic coding of verbal material interfered with visuospatial working memory processing, which probably occurred via the interaction with the executive system due to the natural relevance of verbal stimuli.

On the other hand, the fMRI results showed that the main group differences, regardless of the task, were that the patients with T1D showed less cortical activation than the control subjects. In fact, the main cortical peak observed in the group of T1D patients was located in the right inferior frontal gyrus (orbital area). Moreover, this group showed more subcortical activations, particularly in the cerebellum, insula and the putamen. Previous studies have shown that the insula commonly shows activations during working memory tasks [45]. In particular, the anterior insula engages the brain’s attentional working memory and higher-order control processes while disengaging other systems that are not immediately relevant to the task [54]. Additionally, this model suggests that once a stimulus activates the anterior insula, it will have preferential access to the brain’s attentional and working memory resources. On the other hand, it seems that the left putamen is involved in hindering irrelevant information from entering working memory [34].

Furthermore, the patients with T1D exhibited strong activation within the cerebellum. According to the functions attributed to the cerebellum in working memory [41, 55, 56], there is the general belief that the cerebellum may modulate filtering processes in the basal ganglia via a cortico-cerebellar circuitry [41]. It has also been hypothesized that the cerebellum may operate as a type of internal timing system [57], and it is involved in executive control and inhibition of distractors [41]. Additionally, [1] suggested that the cerebellum is one of the most affected brain structures during neurodevelopment in children with T1D. This finding could explain the activation differences between the groups in our study, as the patients with T1D exhibited more activation in the tonsil of the posterior lobe of the right cerebellum posterior lobe in the contrasts A-B and C-D. Potentially, the cerebellum has adapted over time during neurodevelopment in our sample of T1D subjects, which has been suggested in other studies [32].

It is well known that the right orbital area is critical for successful implementation of inhibitory control [58, 59]. This area is clearly activated in patients with T1D, which suggests that patients with T1D require more attention, monitoring and internal regulation resources than healthy controls, and then, to successfully perform the working memory tasks, the patients recruit other cerebral areas beyond the dorsolateral prefrontal cortex. In this case, the lesser activation of the dorsolateral prefrontal cortex may be related to the involvement of these other prefrontal regions to support working memory processes. In addition, the putamen, insula and the cerebellum could constitute a functional portion of this network, which is responsible for filtering irrelevant information and thus makes the attentional resources available to perform the task. However, when greater executive control resources were needed, the processing efficiency in T1D participants seemed to be diminished, which is probably due to the higher memory and attentional demands [40].

Similar to our results, [60] reported increased BOLD activation in the cerebellum and right frontal pole in a task with a greater cognitive load. Equally important is the fact that [61] found a relative absence of hierarchical high-level hubs in the prefrontal lobe of patients with T1D and suggested that dysfunctional cortical organization underlies the ineffective top-down control of the prefrontal cortex. These findings could provide an explanation for our results regarding the diminished cortical activations and increased subcortical activations.

In accordance with these findings, changes in the brain activation pattern in patients with T1D could be neuroplastic adaptations to frequent glucose dysregulation experiences since the diagnosis [32]. Consequently, the activations in the right inferior frontal area, the cerebellum and the putamen observed in our T1D subjects may be an adaptive response to attain the same level of behavioral performance as the healthy subjects. Other works support the idea that the brains of patients with T1D may develop some adaptations to prevent cognitive problems. From a behavioral point of view, the meta-analyses on the neuropsychological performance of T1D patients suggest that the neuropsychological alterations in this population are limited to some cognitive areas and within a range of mild to moderate severity [10, 11]. Some fMRI studies have also supported this idea. [18] showed that in an n-back task, patients with T1D and retinopathy, compared with patients without, showed less deactivation in the anterior cingulate and the orbital frontal gyrus during hypoglycemia compared with euglycemia. However, both groups performed equally on task accuracy and reaction times. [30] showed a pattern of intrinsic hyperconnectivity in children with T1D with respect to normal children, and the authors found a positive association between high connectivity patterns and good cognitive functioning in children with diabetes. [31] showed a similar pattern of hyperconnectivity in adult patients with T1D without retinopathy to that in patients with diabetes and without vascular damage, and the hyperconnectivity pattern was related to better information processing speed and general cognitive performance. All three works interpreted their findings as brain adaptations to prevent cognitive impairment in T1D.

The present study has several limitations. Most likely, the main limitation is due to the fact that glucose levels and glycated hemoglobin on fasting were not measured in the control group. Due to the lack of antecedents, clinical symptoms of diabetes, or of any related disease, the glucose levels in the control group were presumed to be within the normal limits. Moreover, the study did not evaluate factors such as the evolution of T1D over time. Nevertheless, we studied a very homogeneous T1D sample, since our study was focused on very young patients with early onset of the disease and only a few years of disease evolution, with good general health status and adequate glycemic control. This decision was based on the notion that later T1D onset may be associated with a different pattern of functional neuronal network relationships, which could potentially lead to different behavioral strategies and performance outputs. Therefore, these issues should be explored in future studies. Additionally, we did not measure how the verbal stimuli were encoded. It would have been useful to know the strategy that the subjects used to solve tasks C and D. Furthermore, the small sample size may limit the general implications of our results. Nevertheless, the BOLD activations obtained while performing the tasks were compatible with the brain activation patterns found in other fMRI studies [18, 62].

## Conclusions

In brief, the present results reinforce the notion that T1D impacts brain activity while cognitive abilities are evolving that could potentially trigger different degrees of cognitive disturbance, which has been stated by other authors [11, 63, 64]. However, at the same time, our results also support the idea that under some circumstances the brains of T1D patients may develop some adaptations to prevent cognitive dysfunction, presenting different patterns of brain activity that could permit patients with diabetes to achieve the same levels of cognitive performance as healthy subjects. Other studies have supported this idea [18, 30, 65, 66]. However, more studies are needed to confirm this hypothesis and determine the variables responsible for these adaptations.
